# Adherence to Dihydroartemisinin + Piperaquine Treatment Regimen in Low and High Endemic Areas in Indonesia

**DOI:** 10.1155/2022/4317522

**Published:** 2022-03-11

**Authors:** Irfanul Chakim, Tepanata Pumpaibool, Sayono, Ekha Rifki Fauzi

**Affiliations:** ^1^College of Public Health Sciences, Chulalongkorn University, Institute Building 2-3, Soi Chulalongkorn 62, Phyathai Rd, Pathumwan, Bangkok 10330, Thailand; ^2^Faculty of Public Health, Universitas Muhammadiyah Semarang, Semarang, Indonesia; ^3^Faculty of Science & Technology, Universitas PGRI Yogyakarta, Yogyakarta, Indonesia

## Abstract

After decades of successful artemisinin regimen in combating malaria, its effectiveness has decreased since parasite resistance to the treatment regimen has begun to appear. Adherence to artemisinin combination therapy (ACT) in a population is considered to be the key factor contributing to such resistance phenomenon. Although several studies have tried to demonstrate adherence to several ACT types in a population, only a limited number of studies demonstrated adherence to dihyrdroartemisinin + piperaquine (DHP) regimen. The present study was conducted in two localities representing low and high endemic areas in Indonesia. Active case detection (ACD) and passive case detection (PCD) have been applied to screen for malaria case in the localities. At day 3, patients were visited in the house to be interviewed using structured questionnaire. Capillary sample of each patient was also collected on Whatman® filter paper at day 60 to observe the piperaquine metabolite of the patients. 47 and 91 (out of 62 and 138) patients from Jambi and Sumba, respectively, were successfully enrolled in this study. In Jambi, the level of adherence was 66%, while that in Sumba was 79.1%. The associated factors of adherence in our study settings are patient age group (OR = 1.65 [CI: 0.73–3.73]) and patients' knowledge of malaria prevention measure (OR = 0.29 [CI: 0.09–0.9]). Our study suggested that the adherence to ACT medication among population in our study setting is considered to be less than 80%, which needs to be elevated to avoid the growing trend of treatment failure as seen globally. Additionally, our study found that metabolite at day 60 after prescription of piperaquine could be a potential marker for monitoring adherence to piperaquine drug in a population.

## 1. Background

Artemisinin is a class of antimalarial drugs belonging to a plan species called *Artemisia annua* [1]. After approximately thirty years of its first discovery, WHO recommended the medication of ACT to combat *Plasmodium* malaria which has been resistant to conventional antimalarial drugs [2]. Afterwards, in 2010, majority of the world has applied ACT as first-line treatment against malaria and more than half of countries applied ACT as a free-of-charge medication [3, 4]. ACT is considered fascinating because, in addition to ITN and IRS, it has effectively averted 17–28% of the total 663 million clinical cases [5]. However, after the first introduction of artemisinin-resistant parasites found in Cambodia in 2008, the effectiveness of ACT seems to be worrying [6–9].

Besides the development of genetic factors of the parasite due to continuous exposure from the drug, population adherence to ACT is one of the most important factors facilitating the parasite to develop resistance stage [10–12]. Nonadherence behavior can promote malaria parasite to undergo suboptimal dose of artemisinin and its partner drug and it will eventually become fitter, leaving beneficial genetic variation of the parasite [13, 14]. These resistances have been observed in some parts of the world including Southeast Asia and sub-Saharan Africa [9, 15–22]. In Indonesia, until recently, artemisinin has been proven to be still highly efficacious without any sign of resistance [23, 24]. Although triple artemisinin-based combination therapies and prolonged treatment of artemisinin have been proposed, this may raise obstacle on safety and tolerability as well as more adverse circumstance of nonadherence behavior in a population [25, 26]. In order to prevent such worsening scenario of the spread of resistance to currently available antimalarial drugs, a high level of adherence in a population needs to be strictly monitored and maintained [13, 27].

Several studies have attempted to discover population adherence to ACT medication. Dosing of three-day regimen of AS + SP in Zambia [28] has been known to have 78% of population adherence, while in Uganda it was higher, up to 93% [29]. Contradictory findings have been observed in Malawi regarding ACT adherence, where one study found adherence of <30% [30] and another discovered a 100% adherence level [31]. A very low adherence level has also been reported in the Democratic Republic of the Congo [32]. In contrast, a high adherence level following dosing regimen of AL has been observed in Lao PDR [33] and Burkina Faso [34]. Risk factor of nonadherence behavior seems to vary between studies. Several risk factors have been reported in relation to poor adherence to non-ACT regimen, that is, sex [32], age [35], vomiting [36, 37], and advices from local health workers [34]. Interestingly, only one study examined population adherence to DHP treatment [38, 39]. The study was conducted in northern Ghana and found that the adherence of DHP was only 50.9% [39].

A common method to measure adherence is with either self-report or interview [40]. In the case of ACT, several methods have been used to measure adherence, for example, questionnaires only [28, 30, 34], questionnaires and pill count [29, 32, 33], questionnaires with MEMS (Medication Event Monitoring System) [31], and questionnaires and drug metabolites [29, 41–45]. It was considered that a mere questionnaire may under- or overestimate adherence in a population; thus the use of additional information from MEMS and drug metabolite will be helpful for obtaining conclusive finding [46]. Several studies have tried to discover adherence by using drug metabolite, but it was only limited to lumefantrine drug [29, 41–45]. No study has ever demonstrated the use of drug metabolite for measuring adherence to piperaquine as partner drug of artemisinin. It is hypothesized that the drug concentration of piperaquine on day 60 may indicate adherence and nonadherence behavior [46]. Piperaquine metabolite is still in a measurable amount until day 63. Several pooled analysis studies indicate that incomplete DHP prescription results in lower amount of piperaquine metabolite which is measurable at day 60 [46].

In Indonesia, ACT has been introduced as the first-line treatment against malaria parasites. However, after a decade of utilization, no study has ever been conducted to discover population adherence to ACT medication in Indonesia. It is imperative to strictly monitor population adherence to ACT in Indonesia, since neighboring countries of Indonesia, that is, Thailand, Vietnam, and Cambodia, have observed a significant development of the parasite resistance to ACT medication. Additionally, our study, which involved a piperaquine metabolite quantification, can help scientific community to conduct further research by using piperaquine metabolite, where piperaquine has been used as partner drug of artemisinin. Information presented herein will help policymakers to consider the use of day-60 piperaquine metabolite combined with a structured questionnaire for monitoring population adherence to ACT to prevent the development of parasite resistance.

## 2. Method

### 2.1. Study Setting

This study used an observational design with follow-up following the completion day of DHP medication. The study was conducted between January and December 2018 on patients treated with DHP in two different localities representing low and high endemic areas in western and eastern parts of Indonesia. The first sampling area was Lembah Masurai subdistrict in Jambi Province, which is densely forested area located in western part of Indonesia. The second locality was Sumba Island, Nusa Tenggara Timur Province, which has a relatively low vegetation cover and is located in eastern part of Indonesia. Jambi Province had an annual parasite index that varied from 0.05 to 0.14, while the index in Nusa Tenggara Timur Province was varied from 5.41 to 5.76 between 2016 and 2017 [47].

Malaria was a common disease in the areas. In Lembah Masurai subdistrict, malaria was dominated with *Plasmodium vivax* with limited number of *Plasmodium falciparum* found. Meanwhile, in Sumba Island, three of the five known malaria parasites in Indonesia, that is, *Plasmodium falciparum*, *Plasmodium vivax*, and *Plasmodium malariae*, were commonly found. DHP was distributed as a free-of-charge medication for malaria by local health facilities. Every malaria case in the area was treated with DHP treatment according to the Ministry of Health of the Republic of Indonesia. People aged 0–11 months with body weight between <4 and 10 kg were treated with ½ tablet of DHP, while people aged 1–4 years with body weight of 11–17 kg were given 1 tablet; further, people aged 5–9 years (18–30 kg), 10–14 years (31–40 kg), >15 years (41–59 kg), and >15 years (>60 kg) were given 1 ½, 2, 3, and 4 tablets, respectively [48].

### 2.2. Sample Size

The estimation of sample size was based on the formula *n*=*z*^2^*P*(1 − *P*)/*d*^2^, where *Z*^2^ is the level of confidence at 99%, *d*^2^ is the 4% precision, and *P* is the following assumed adherence level. We assumed that the level of adherence in the population is 70%. With addition of 10% for contingencies, the minimum sample is 138.

### 2.3. Recruitment and Data Collection Method

Initially, active and passive case detection was carried out to detect any malaria case in the area. ACD was performed to those who had fever >37.5°C. PCD was implemented by local health worker on those who visited the local health care center with suspected clinical sign and symptom related to malaria. Laboratory performance was carried out by collecting capillary sample on slide glass, which was detected under light microscope. The person who tested positive for any *Plasmodium* malaria was immediately prescribed a standard dose of DHP as mentioned above. There were two types of questionnaires in our study based on previously published paper with minor modification [32].

#### 2.3.1. Center Questionnaire

All people who tested positive for *Plasmodium* malaria were then treated with standard DHP treatment either in the screening site or in the local health center. The questionnaire was obtained from the local health facility in the case of PCD and the screening site in the case of ACD. At the time after prescription (day 0), all patients were interviewed using center questionnaire containing patient/caregiver details including name, age, sex, the number and type of prescriptions, and information regarding the understanding of patient/caregiver towards ACT and pharmacy dispensing practices. Center questionnaire was performed by the researchers in the case of ACD and by local health staff in the case of PCD. The center questionnaire can be downloaded from Supplementary Material 1.

#### 2.3.2. Home Questionnaire

After the completion day of DHP medication (day 3), patients were visited to have “home questionnaire” interview. There was no information given to the patients after filling in the center questionnaire about the upcoming home questionnaire to avoid behavioral bias. It specifically assessed the adherence of the patient to DHP medication. Any sociodemographic characteristic of the patients/caregivers was explored at this stage, followed by a systematic question of how pills were taken. Besides the answer of each patient/caregiver, blister package was observed to find whether the pills were taken correctly or if any remaining pills were found. Any reason for not complying with the treatment regimen was recorded. There were some additional questions to assess patient's/caregiver's understanding about knowledge of malaria cause and prevention. Any patient who was not getting better after treatment has been referred back to the local health facility. Home questionnaire was performed entirely by the researchers. The home questionnaire can be downloaded from Supplementary Material 2.

The definition of adherence was following the previous paper [32]. Adherence was defined by either the answer of the patient/caregiver or the presence of any DHP pill inside the blister package. Accordingly, there are 3 classifications of adherence: certain nonadherence, that is, when the remaining DHP pills have been seen; probable nonadherence, that is, if the blister pack is empty and patient/caregiver has given incorrect answer regarding the necessary intake (pill count or time schedule); and probable adherence, that is, if the blister pack is empty and patient/caregiver has given correct answer regarding the necessary intake (pill count and time schedule).

### 2.4. Piperaquine Blood Metabolite

Capillary samples were collected from all patients at the same time when performing the home questionnaire. The capillary sample of each patient was collected on Whatman® filter paper at day 60 after the day of prescription (day 0) [46]. Each filter paper was then labeled based on the patient's category: vomiting, certain nonadherence, probable nonadherence, and probable adherence. All the collected capillary samples were sent to Pharmacology Department, Faculty of Tropical Medicine, Mahidol University, Thailand.

### 2.5. Ethical Consideration

Informed consent was obtained from all of the patients or caregivers in case of child participation in this study. Ethical approval was obtained from Hasanuddin University, Indonesia. Our study has sought permission from local health center, Provincial Public Health Office, and the Ministry of Health of the Republic of Indonesia.

### 2.6. Data Management and Statistical Analysis

After completion of all data of the patient, the data were entered into EpiData 3.1 software. Descriptive statistic was used to analyze data on sociodemographic characteristics, percentage of adherence, percentage of reasons for incomplete treatment, and knowledge of the causes and prevention of malaria. Univariate and multivariate analyses were performed to associate factors related to nonadherence behavior with ACT. Univariate and multivariate analyses were performed with SPSS version 22.0 (IBM Corp, Armonk, NY, USA). To estimate odds ratio, we used Review Manager (RevMan) version 5.3, Cochrane. Visualization was performed using GraphPad Prism version 7.00 for Mac (GraphPad Software, La Jolla, California, USA).

## 3. Result

### 3.1. Survey Profile

In total, 200 patients tested positive for malaria parasite and were given DHP medication. Out of those 200 patients, 62 patients were detected in Jambi Province and the other 138 were from Sumba Island. In Jambi, out of 62 malaria patients, 47 (75.8%) patients were able to be visited and interviewed at the day of completion of the medication. The remaining 15 (24.2%) patients were unable to be visited because they traveled outside the study area. In Sumba Island, 138 patients were given DHP treatment. However, only 91 patients (65.9%) were successfully collected for home-visit interview. The remaining 47 patients (34.1%) were unable to reach because of either working inside forestry area or traveling to unknown area. No patient has ever refused to be our study participant.

### 3.2. Sociodemographic Description

In Jambi, the majority of study participants were adults (19/40.4%) and adolescents (18/38.3%), while the rest were young children (10/21.3%). On the other hand, the participants in Sumba Island were dominated by adolescents (39/42.9%) and young children (30/33%), while the rest were adults (20/22%) and infants (2/2.2%). Sex ratio in Jambi was 1.8 (male/female: 30/17), while that in Sumba Island was 1.3 (male/female: 52/39). In Jambi, the majority of the patients were uneducated (24/52.2%; illiterate and not completing primary school), while the rest of them were poorly educated (18/39.1%; completed primary education, did not complete secondary education, and completed secondary education) and highly educated (4/8.7%; did not complete higher education and completed higher education). Similar condition has been observed in Sumba, where the majority of the patients were uneducated (69/75.8%; illiterate and not completing primary school), and the rest of them were poorly educated (18/19.8%) and highly educated (4/4.4%). Regarding caregiver education, it was the opposite, where Jambi was dominated by poor people and highly educated people (93.1%), while Sumba Island was dominated by uneducated and poor population (93.4%) (Table 1).

In Jambi, most household sizes were proportional (1–4 household members; 76.6%), while Sumba Island was dominated by nonproportional household sizes (5–8 household members; 72.5%) (Table 2). In Jambi, 38.3% of the households had one child below five years of age and 21.3% had two children below five years of age. The rest of the patients (40.4%) had no children below five years of age. Contrarily, all households had at least 1 child below five years of age in Sumba (range: 1–6). Jambi and Sumba Island shared a similar pattern of profession of heads of households, which is farmer (Jambi: 95.7%; Sumba: 75.8%).

### 3.3. Patient Adherence

In Jambi, there was one out of 47 (2.1%) patients whose pills were visible or whose tablets remained in the blister package at the day of completion of DHP medication (Table 3). 15 out of 47 (31.9%) patients were considered to have probable nonadherence, since no blister was seen and they answered incorrectly about the dosage they should have taken. The remaining 31 (out of 47) patients (66%) were considered to have probable adherence, since no blister was seen and the patients answered correctly about the dosage they should have taken. Additionally, out of those patients with probable adherence, two (6.5%) (out of 31) had no pills inside the blister package with correct answer. In Sumba Island, two (2.2%) of the total interviewed patients (out of 91) still had DHP pills in their blister package and were considered to have certain nonadherence. 17 patients (out of 91) were considered to have probable nonadherence, since no blister was seen and they have incorrectly answered about the dosage they should have taken. The remaining 72 patients (79.1%) were categorized as having probable adherence (no blister was seen and they gave correct answer about dosage). Two patients from Jambi and four patients from Sumba Island vomited the pills. In detail, one patient from Jambi vomited the pills on day 1 and day 2 but continued to take the drug on day 3 without vomiting. The second patient vomited the pills on day 1 but continued to take the drug on day 2 and day 3. On the other hand, three patients from Sumba vomited the pills on day 1 but continued the medication on day 2 and day 3. One patient vomited the pills on day 1 and day 2 but continued for the rest of the medication course.

### 3.4. Reason for Incomplete, Incorrect, and Correct Intake

Patient-reported reasons for incomplete, incorrect, and correct medication intake were recorded (Table 4). In Jambi, the reason for incomplete medication intake was that the patient was cured and did not need to continue the medication, while in Sumba, the reason was that the patient forgot to take the pill or the caregiver forgot to give them the pill, as well as other reasons. The major reason for incorrect intake of ACT medication was similar in Jambi and Sumba, which is patient/caregiver claimed that incorrect instruction was given. Similarly, the reason for correct intake in the two localities was that correct instruction was given in the clinic, primary health facility, or sampling location (88% in Jambi and 98.6% in Sumba). However, six patients' (12.8%) data from Jambi were missing for reasons given for correct intake.

### 3.5. Assessment of Possible Risk Factors

Univariate and multivariate analyses for assessing possible risk factors for nonadherence behavior have been done (Table 5). The possible risk factors included sex, patient age group, education of caregiver, understanding of the causes and prevention of malaria, including bed nets that can prevent malaria and the presence of bed nets inside household, and the understanding of ACT use. Age group was associated with nonadherence behavior (*P* value = 0.023). Infants and young children were more likely to be nonadherent to ACT medication (OR = 1.65 [CI: 0.73–3.73]). The other risk factor of nonadherence behavior was the patient's understanding of malaria cause and prevention and the fact that bed net prevents malaria (*P* value = 0.032). The patient who had no understanding of malaria cause and prevention was more likely to be nonadherent (OR = 3.42 [CI: 1.108–10.6]).

### 3.6. Analysis of Piperaquine Metabolite

**Figure 1:**
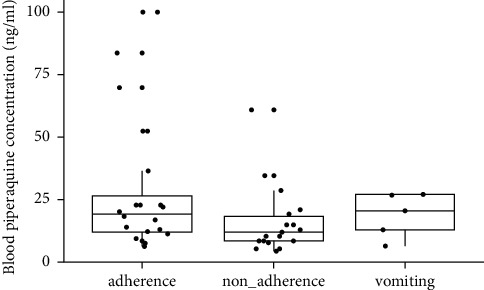
Box plots of blood piperaquine concentrations by adherence types (adherence, nonadherence, and vomiting). Horizontal line in the boxes represents median, while lower and upper error bars represent the first and the third quartiles.

We only managed to measure piperaquine metabolite at day 60 in 48 samples due to insufficient amount of capillary blood volume taken from the rest of the samples. The overall median piperaquine level at day 60 was 14.9 *μ*g/mL (min–max: 4.71–100 *μ*g/mL; mean: 22.4 *μ*g/mL). The measurement of piperaquine metabolite was differentiated into three groups: adherence, vomiting, and nonadherence. A wide variation of individual median piperaquine level can be seen within each group (Figure 1). Median piperaquine levels in adherence and vomiting groups were higher than that in nonadherence group (19.4 *μ*g/mL versus 11.9 *μ*g/mL and 20.6 *μ*g/mL versus 11.9 *μ*g/mL, respectively). The reason why the vomiting group has higher median piperaquine levels compared to the nonadherence group might be because the vomiting group continued to take the medication course after vomiting but the nonadherence group took medication on only one or two days.

## 4. Discussion

It was widely known that poor adherence of a population to antimalarial medication will lead to the development of treatment failure due to the spread of genetic resistance in parasites [11, 49, 50]. There are several risk factors that are known to promote the emergence of malaria parasite, that is, population coverage for antimalaria medication, half-life of the selected drug, the residue of the drug inside host body, a high mutation rate of the parasite, fitness of early developed resistant parasite population, declining transmission intensity, and a low coverage for other preventive measures [51]. Interestingly, by maintaining good quality ACT, either pharmacokinetically or by improving population compliance, it is possible to eliminate malaria even in the area where ACT resistance has been spread [52]. It was also previously described that a higher rate of treatment failure occurred when adherence level was lower compared to optimal adherence level [53]. It was shown that the probability of treatment failure was about 4 times higher each time the patient missed the dose [53]. In fact, although full adherence has been achieved, it still leads to ∼5% of treatment failure in the patients [53].

Our study shows that the levels of adherence to ACT medication among population in Indonesia are 66% (Jambi) and 79.1% (Sumba). If several studies on ACT adherence are accumulated, the average adherence level is 75.2% [29, 30, 36, 37, 41, 44, 45, 54–75]. Therefore, it can be said that adherence to ACT among population in Jambi Province is below the average level of adherence to ACT worldwide (66% versus 75.2%), while in Sumba, the adherence level is only slightly higher than the average (79.1% versus 75.2%). However, none of the studies have discovered adherence to DHP medication among the general population [29, 30, 36, 37, 41, 44, 45, 54–75]. Only one study has described the adherence to DHP medication among population in Northern Ghana, the result of which was 50.9% of adherence lower than our present study [39]. In fact, it is difficult to have conclusive finding regarding adherence to ACT medication at the global population level due to the fact of varied study design, study protocol, and ACT prescription type. Taken together, the adherence to ACT medication among population in our study setting is considered to be less than 80%, which needs to be elevated to avoid the growing trend of treatment failure as seen globally [9, 15–22].

The main reason for correct intake of ACT in our study is similar to that in the previous study, which is that a correct instruction has been given in the local health facility or clinic [32]. The other patients claimed that they have taken the same pills before as recognition of the drug is an imminent factor in adherence to the treatment regimen. The reason for nonadherence behavior in our study is seemingly similar to the other findings. The reason for certain nonadherence (where there were still pills left) was that patients felt better or forgot to take their medication and those were the usual reasons for them not to take proper medication, as was the case in the other findings [37, 55, 57, 76]. Similarly, the main reason for probable nonadherence was that the patients claimed that an incorrect instruction has been given in the local health facility or clinic, and it may be because they lack understanding of the prescribed drug [55, 76]. Pharmacist needs to give this particular type of patient more detailed explanation of the drug and how to take it properly. Additionally, some patients explained that the reason why they had probable nonadherence was that they thought that taking all the pills on the first or second day of treatment will cure them faster or because patients could not swallow the pills or other reasons. Such reasons are generally found throughout studies [36, 37, 57, 59] and the importance of targeted health promotion to improve patient's awareness of the impact of improper adherence behavior to the treatment regimen was emphasized.

Factors associated with adherence to ACT are varied between studies [29, 36, 37, 44, 45, 55, 57, 58, 60, 62, 65, 66, 69, 70, 75]. The factors associated with adherence in current study were patient age group (OR = 1.65 [CI: 0.73–3.73]) and patients' knowledge of malaria prevention (OR = 3.42 [CI: 1.108–10.6]). Age has been known to be associated with adherence to ACT. Our finding is similar to that in the work of Mace et al. [58], where the younger the person was, the more likely they were to be nonadherent to ACT medication. As opposed to previous findings, Lawford et al. [60] found that the older the person was, the more likely they were to be nonadherent. It was postulated that the older the patient, the better their understanding of ACT administration and they may have had prior experience in taking the treatment [35, 60]. It has been also previously found that lack of appropriate dose formulation may lead to improper adherence behavior in such age group [77]. It is one of the concerned problems that the development of parasite resistance is higher in children because higher parasite biomass inside them increases the possibility of developing *de novo* resistance of the parasite [65, 77, 78]. Another risk factor discovered in our study is patient's understanding of malaria prevention strategy (the use of bed nets). A slightly different finding has been discovered by Gerstl et al. [36], where adherence to ACT has been associated with patient's recognition of malaria cause (malaria transmitted by mosquito bites) rather than malaria prevention strategy. Taken together, it is imperative to monitor adherence especially in infants and young children because such vulnerable age group is intensifying the development of parasite resistance. Additionally, public policymakers need to consider promoting a better understanding of malaria cause and prevention starting from the local health workers and eventually down to local society.

One of the challenging issues regarding adherence measurement is to set up a precise method rather than questionnaire which may contain social desirability bias [28–30, 32–34, 41–46]. In terms of adherence to antimalarial drug, the only available metabolite measurement is limited to lumefantrine [29, 41–45]. We tried to prove previously hypothesized technical method to measure piperaquine adherence using pharmacokinetic approach in a population which stated that day 60 after initial prescription could be a marker for piperaquine adherence [46]. It has been reviewed previously that piperaquine metabolite is still measurable until day 63 even in children [46]. After achieving sufficient concentration up to day 3 during treatment course, piperaquine metabolite will decrease slowly and linearly until it reaches observable limit at day 63 [46]. We found that median piperaquine level from adherence group was higher compared to that in nonadherence group. This finding indicated that piperaquine measurement at day 60 is a novel assessment that has the potential to be a monitoring tool for adherence to piperaquine. Further study needs to be conducted with significantly higher sample size to better evaluate the threshold of piperaquine metabolite between adherent and nonadherent individuals. However, metabolite measurement at day 60 could lead to sampling issue. As seen in our result, the number of patients who were successfully taken for capillary sample was reduced significantly due to technical reasons, for example, unreachable residence location, losing contact with patients, and unwillingness to participate. This drawback can be reduced by carefully selecting patient who is more accessible or providing technical support in order to reach out gathering spot for specific population of patients.

## 5. Conclusion

After decades of the implementation of ACT as the official first-line treatment for malaria, ACT now seems to be less effective, since parasites that are resistant to ACT have been observed globally. One of the imminent factors contributing to the development of such resistance is nonadherence behavior to ACT treatment regimen. A plenty of studies have described adherence to several artemisinin combination therapies among population, but specific studies examining adherence to DHP are limited. Our study presented adherence to DHP as current artemisinin combination therapy in population in two localities representing low and high endemic areas in Indonesia. Our study found nonsatisfying level of adherence in the localities. The factors associated with adherence in our study setting were age and understanding of malaria prevention strategy. The present study clearly demonstrated the need for more careful monitoring of adherence level in a population. Additionally, day 60 after prescription of piperaquine metabolite can be beneficially combined with a structured questionnaire to assess adherence in a population.

## Figures and Tables

**Table 1:** The description of sociodemographic variables of the patients and the caregivers in Jambi and Sumba.

Sociodemographic factor	Jambi (%)	Sumba (%)

Age group		
<1 (infants)	0	2 (2.2)
2–5 (young children)	10 (21.3)	30 (33)
6–13 (adolescents)	18 (38.3)	39 (42.9)
>14 (adults)	19 (40.4)	20 (22)
Total	47 (100)	91 (100)
Sex		
Male	30 (63.8)	52 (57.1)
Female	17 (36.2)	39 (42.9)
Total	47 (100)	91 (100)
Caregiver's relation to patient		
Patient	18 (38.3)	30 (33)
Father/mother	25 (53.2)	54 (59.3)
Grandfather/grandmother	1 (2.1)	2 (2.2)
Brother/sister	2 (4.3)	3 (3.3)
Uncle/aunt	1 (2.1)	2 (2.2)
Total	47 (100)	91 (100)
Educational attainment of patient		
Illiterate	8 (17.4)	51 (56)
Did not complete primary education	16 (34.8)	18 (19.8)
Completed primary education	13 (27.7)	12 (13.2)
Did not complete secondary education	1 (2.2)	0
Completed secondary education	4 (8.7)	6 (6.6)
Did not complete higher education	0	0
Completed higher education	4 (8.7)	4 (4.4)
Total	46 (100)	91 (100)
Educational attainment of caregiver		
Illiterate	1 (3.4)	21 (34.4)
Did not complete primary education	1 (3.4)	13 (21.3)
Completed primary education	14 (48.3)	13 (21.3)
Did not complete secondary education	0	4 (6.6)
Completed secondary education	4 (13.8)	6 (9.8)
Did not complete higher education	0	0
Completed higher education	9 (31)	4 (6.6)
Total	29 (100)	61 (100)

**Table 2:** The description of sociodemographic information of household in Jambi and Sumba.

Sociodemographic factor of household	Jambi (%)	Sumba (%)

Number of household members		
1–4	36 (76.6)	21 (23.1)
5–8	10 (21.3)	66 (72.5)
9–12	1 (2.1)	4 (4.4)
Total	47 (100)	91 (100)
Number of children in household (below five years of age)		
0 children	19 (40.4)	0
1 child	18 (38.3)	47 (51.6)
2 children	10 (21.3)	15 (16.5)
3 children	0	3 (3.3)
4 children	0	1 (1.1)
5 children	0	0
6 children	0	1 (1.1)
Total	47 (100)	67 (100)
Profession of head of household		
Farmer	45 (95.7)	69 (75.8)
Trader	1 (2.1)	0
Employee	0	2 (2.2)
Odd jobs	0	1 (1.1)
Unemployed	0	10 (11)
Others	1 (2.1)	5 (5.5)
Total	47 (100)	87 (100)

**Table 3:** Adherence to DHs regimen among population in Jambi Province and Sumba Island, Indonesia.

	Jambi		Sumba	
Calculation of adherence	Incomplete/incorrect intake described	Complete/correct intake described	Incomplete/incorrect intake described	Complete/correct intake described

No blister	15	25	17	68
Empty blister pack	0	6	0	4
Blister pack with pills	1	0	2	0
Total	16	31	19	72
Classification of adherence	Number of patients	Proportion (%)	Number of patients	Proportion (%)
Certain nonadherence	1	2.1	2	2.2
Probable nonadherence	15	31.9	17	18.7
Probable adherence	31	66	72	79.1
Total	47	100	91	100
Adherence status	Number of patients	Proportion (%)	Number of patients	Proportion (%)
Nonadherent	16	34	19	20.9
Adherent	31	66	72	79.1
Total	47	100	91	100

**Table 4:** Reason for incomplete, incorrect, and correct intake given by the patients.

	Jambi		Sumba	

Reasons given for incomplete intake (pills remaining)	*N*	Percentage (%)	*N*	Percentage (%)
Patient was cured and did not need to continue the medication	1	100	0	0
Patient was cured and saved the pills for other occasions	0	0	0	0
The household members are poor and saved the pills for other occasions	0	0	0	0
Patient forgot to take the pills/caregiver forgot to give the pills	0	0	1	50
Patient felt unwell/the medication was not working properly	0	0	0	0
Patient/caregiver claims that incorrect instruction was given	0	0	0	0
Others	0	0	1	50
Total	1	100	1	100
Reasons given for incorrect intake	*N*	Percentage	*N*	Percentage
Patient/caregiver thought that the patient will cure faster	1	6.7	0	0
Patient/caregiver claims that incorrect instruction was given	13	86.7	15	88.2
The pills given made the patient feel sick/unwell	0	0	0	0
Patient cannot swallow the pills	1	6.7	0	0
Patient was vomiting	0	0	0	0
Others	0	0	2	11.8
Total	15	100	17	100
Reasons given for correct intake	*N*	Percentage	*N*	Percentage
Patient/caregiver/household member has taken the same pills before, so they understood how to take them	2	8	1	1.4
Correct instruction was given in the clinic/primary health facility/sampling location	22	88	71	98.6
Patient was helped by local community health volunteers	0	0	0	0
Others	1	4	0	0
Total	25	100	72	100

**Table 5:** Associated risk factors of adherence to DHP medication in Indonesia

Risk factors	Adherence	Nonadherence	OR	95% CI	*P* value

Sex					
Male	62	20	0.882	0.405–1.918	0.751
Female	41	15			
Total	103	35			
Age group					
Infants and young children	26	16	1.65	0.73–3.73	**0.023**
Adolescents and adults	77	19			
Total	103	35			
Education attainment of caregiver					
Illiterate	25	11	1.26	0.49–3.20	0.631
Any education	40	14			
Total	65	25			
Having knowledge of the fact that bed net prevents malaria					
Yes	96	28	3.42	1.108–10.6	**0.032**
No	7	7			
Total	103	35			
Bed nets observed					
Yes	98	32	1.53	0.27–8.76	0.63
No	4	2			
Total	102	34			
Understanding of ACT use					
Yes	21	7	1.02	0.39–2.67	0.961
No	82	28			
Total	103	35			

## Data Availability

The final analysis data used to support the findings of this study are included within the article.

## Abbreviations

DHP: Dihydroartemisinin + piperaquine
ACT: Artemisinin combination therapy
ACD: Active case detection
PCD: Passive case detection
OR: Odds ratio
CI: Confidence interval
WHO: World Health Organization
ITNs: Insecticide treated bed nets
IRS: Indoor residual spraying
AS: Artesunate
SP: Sulfadoxine-pyrimethamine
AL: Artemether-lumefantrine
MEMS: Medication Event Monitoring System
kg: Kilogram.

## Supplementary Materials

Supplementary Materials The center questionnaire can be downloaded from Supplementary Material 1. The home questionnaire can be downloaded from Supplementary Material 2.
