# Supplementary material for: Adherence to Dihydroartemisinin + Piperaquine Treatment Regimen in Low and High Endemic Areas in Indonesia
Source: J Trop Med. 2022 Mar 11;2022:4317522. doi: 10.1155/2022/4317522 (PMC8933069; doi:10.1155/2022/4317522)
Supplement: Supplementary Materials — The center questionnaire can be downloaded from Supplementary Material 1. The home questionnaire can be downloaded from Supplementary Material 2. [file 4317522.f1.zip › 4317522.f1/Supplementary 1- Center questionnaire.docx]

| **DHP ADHERENCE STUDY, INDONESIA** | | | | | | | | | | | | | | | |  |  |  |  |
| --- | --- | --- | --- | --- | --- | --- | --- | --- | --- | --- | --- | --- | --- | --- | --- | --- | --- | --- | --- |
|  | |  | |  |  |  |  | |  |  | | |  |  | |  |  |  |  |
| **CENTRE QUESTIONNAIRE at health Centers OR screening sites** | | | | | | | | | | | | |  |  | |  |  |  |  |
|  | |  | |  |  |  |  | |  |  | | |  |  | |  |  |  |  |
|  | |  | |  |  |  |  | |  |  | | |  |  | |  |  |  |  |
|  | |  | |  |  |  |  | |  |  | | |  |  | |  |  |  |  |
| Date: \|__\|__\| (dd) / \|__\|__\| (mm) / 2018 | | | | | | | | |  | **Inclusion Nr: HC - \|__\|__\|__\|__¦** | | | | | |  |  |  |  |
|  | |  | |  |  |  |  | |  | *Note: first digit is always the unique number of the data collector* | | | | | |  |  |  |  |
| Name of data collector: ____________________________________ | | | | | | | | | | | | | |  | |  |  |  |  |
|  | |  | |  |  |  |  | |  |  | | |  |  | |  |  |  |  |
|  | |  | |  |  |  |  | |  |  | | |  |  | |  |  |  |  |
| PATIENT'S IDENTIFICATION | | | | | | |  | |  |  | | |  |  | |  |  |  |  |
|  | |  | |  |  |  |  | |  |  | | |  |  | |  |  |  |  |
| Family name: _________________________ | | | | | | | | | Given name: ___________________________________ | | | | | | |  |  |  |  |
|  | |  | |  |  |  |  | |  |  | | |  |  | |  |  |  |  |
| Gender: M / F | | | |  | Age: \|__\|__\| *year(s) or if < 1 year \|__\|__\| month(s)* | | | | | | | | | Weight: _____ kg | |  |  |  |  |
|  | |  | |  |  |  |  | |  |  | | |  |  | |  |  |  |  |
|  | |  | |  |  |  |  | |  |  | | |  |  | |  |  |  |  |
| PATIENT'S ADDRESS *(if needed, you can make a drawing on the back of this page)* | | | | | | | | | | | | | | | |  |  |  |  |
|  | |  | |  |  |  |  | |  |  | | |  |  | |  |  |  |  |
| Family and given name parent/caretaker *(if patient is a child)*: _________________________________ | | | | | | | | | | | | | | | |  |  |  |  |
| Village name: _______________________________________________________________________ | | | | | | | | | | | | | | | |  |  |  |  |
| Section of quartier: ____________________________________________________________________ | | | | | | | | | | | | | | | |  |  |  |  |
| Name of house owner: ________________________________________________________________ | | | | | | | | | | | | | | | |  |  |  |  |
| How many minutes did you walk from home to the health clinic? \|__\|__\|__\| *minutes* | | | | | | | | | | | | | | | |  |  |  |  |
| Other indicators: _____________________________________________________________________ | | | | | | | | | | | | | | | |  |  |  |  |
|  | |  | |  |  |  |  | |  |  | | |  |  | |  |  |  |  |
|  | |  | |  |  |  |  | |  |  | | |  |  | |  |  |  |  |
| BEFORE PATIENT CAME TO THE HEALTH CENTRE | | | | | | | | | | | | | |  | |  |  |  |  |
|  | |  | |  |  |  |  | |  |  | | |  |  | |  |  |  |  |
| How many days ago did the patient's symptoms start? | | | | | | | | | | | | |  | \|__\|__\| *days* | |  |  |  |  |
|  | |  | |  |  |  |  | |  |  | | |  |  | |  |  |  |  |
| Did the patient take any treatment since then? | | | | | | | | | | | | |  | Yes / No | |  |  |  |  |
|  | |  | |  |  |  |  | |  |  | | |  |  | |  |  |  |  |
| If yes, which one/s *(specify)*: ________________________________________________________ | | | | | | | | | | | | | | | |  |  |  |  |
|  | |  | |  |  |  |  | |  |  | | |  |  | |  |  |  |  |
|  | |  | |  |  |  |  | |  |  | | |  |  | |  |  |  |  |
| CARETAKER'S IDENTIFICATION (If only the patient was unable to take the drug by him-/her-self) | | | | | | | | | | | | | | | | | | | |
| Relation to patient | | | | | | | | | Patient is respondent (Adult)  Parent (Mother/Father)  Grandparent (Grandfather/Grandmother)  Sibling (Brother/Sister)  Aunt/Uncle  Other (*specify below*)  *____________________________________________________* | | | | | | | | | | 1  2  3  4  5  6 |
| Caretaker: Gender: M / F Age \|__\|__\| years Adult patient, no caretaker | | | | | | | | | | | | | | | | | | | |
| Highest level of education  *Ask if able to read / write first:*   - *if "no" circle 1 and continue to next question,* - *if "yes" determine what level.* | | | | | | | | | Unable to read and write  Primary but incomplete  Primary completed  Secondary but incomplete  Secondary completed  Higher level but incomplete  Higher level completed | | | | | | | | | | 1  2  3  4  5  6  7 |
| QUESTIONS TO PARENT / CARETAKER | | | | | | | | | | | | | | | | | | | |
| Q1 | | Can you tell me what disease you / your child/ your relative has at that time? | | | | | | | | | | No (Don't know)  Yes – name is Malaria *Signs and symptoms (malaria) only, does not know name* Other *(specify below)*  *______________________________________________* | | | | | | 0  1  2  3 | |
| Q2 | | *Ask to see medicines given by the clinic.*  You / your child/ your relative has malaria. Of the treatments you have here, can you show me which one(s) is for malaria?  *If answers (1) or (4) skip to Q4*  *If answers (2) or (3) continue to Q3* | | | | | | | | | | Yes – Shows ACT only  Shows ACT with other drugs  Shows other drugs only  Doesn't know | | | | | | 1  2  3  4 | |
| Q3 | | Which other drugs shown? Multiple answers possible | | | | | | | | | | Paracetamol (PCM)  Folic acid (FA)  Other unrelated (*specify below)*  *_________________________________________* | | | | | | 1  2  3 | |
| Q4 | | Indicating the ACT tablets Have you or your child/ your relative taken this treatment before?  If yes, how many times? | | | | | | | | | | Yes, ______________________times  No, this is the first time | | | | | | 1  0 | |
| Q5 | | Indicating ACT tablets again: Can you tell me how did you take / to give this treatment?  *Let patient/caretaker enough time to answer, don’t push.*  *____________________________________________________________________________________________*  *____________________________________________________________________________________________*  *____________________________________________________________________________________________*  *____________________________________________________________________________________________*  *____________________________________________________________________________________________*  *____________________________________________________________________________________________*  *____________________________________________________________________________________________*  *____________________________________________________________________________________________*  *____________________________________________________________________________________________*  *____________________________________________________________________________________________*  *___________________________________________________________________________________________* | | | | | | | | | | | | | | | | | |
| ***Clarification of treatment instructions:*** | | | | | | | | | | | | | | | | | | | |
| Q6 | | | | Did the person who gave you this treatment ask if you understood how to give it? | | | | | | | | | Yes  No | | | | | 1  0 | |
| Q7 | | | | Did they ask you to repeat back the instructions to you? | | | | | | | | | Yes  No | | | | | 1  0 | |
| Q8 | | | | Did they give you any other information or advice about the treatment? | | | | | | | | | Yes *(specify below)*  _________________________________  _________________________________  _________________________________  No  No | | | | | 1  0 | |
| Q9 | | | | Did you/your child already/ your relative take one dose of this treatment at the clinic? | | | | | | | | | Yes  No | | | | | 1  0 | |
| Q10 | | | | Did you/your child/ your relative take any other treatment in the clinic?  *If YES, go to Q11* *If NO, go to Q12* | | | | | | | | | Yes  No | | | | | 1  0 | |
| Q11 | | | | If yes, which one? | | | | | | | | | Paracetamol (PCM)  Folic Acid (FA)  Other/s *(specify below)*  Don't Know | | | | | 1  2  3  4 | |
| Q12 | | | | Show the ACT tablets again How many time you need to give/take this treatment after came to the clinic?  *Possible answers: 1 time, 2 times, 3 times, don’t know etc.* | | | | | | | | | ___________________________________  ___________________________________  ___________________________________  ___________________________________ | | | | | | |
| Q13 | | | | How many tablets you need to give / take? | | | | | | | | | \|__\|__\|tablets | | | | | | |
| Q14 | | | | If balance remaining:  What will you do with the remaining tablets? | | | | | | | | | Keep for next time household member unwell  Other *(specify)*_________________________  _____________________________________ | | | 1  2 | | | |
| Q15 | | | | Will you still give/take the treatment after your child/ your relative/you felt better? *(Day 1)* | | | | | | | | | Yes  No | | | 1  0 | | | |
| Q16 | | | | What you need to do If you / your child/ your relative is not better in 3 days? | | | | | | | | | __________________________________  ___________________________________ | | | | | | |

| TREATMENT PRESCRIBED TO PATIENT AT THE HEALTH CENTRE OR THE SCREENING SITE | | | | | | | | | |
| --- | --- | --- | --- | --- | --- | --- | --- | --- | --- |
|  |  |  |  |  |  |  |  |  |  |
| Nr. | Name of treatment prescribed | | | | |  | Nr. of tablets | Times per day | Nr. of days |
|  |  |  |  |  |  |  |  |  |  |
| 1. |  |  |  |  |  |  | \|__\|__\| | OD / BD / TDS | for \|__\|__\| days |
| 2. |  |  |  |  |  |  | \|__\|__\| | OD / BD / TDS | for \|__\|__\| days |
| 3. |  |  |  |  |  |  | \|__\|__\| | OD / BD / TDS | for \|__\|__\| days |
| 4. |  |  |  |  |  |  | \|__\|__\| | OD / BD / TDS | for \|__\|__\| days |
| 5. |  |  |  |  |  |  | \|__\|__\| | OD / BD / TDS | for \|__\|__\| days |
| 6. |  |  |  |  |  |  | \|__\|__\| | OD / BD / TDS | for \|__\|__\| days |
| 7. |  |  |  |  |  |  | \|__\|__\| | OD / BD / TDS | for \|__\|__\| days |
| 8. |  |  |  |  |  |  | \|__\|__\| | OD / BD / TDS | for \|__\|__\| days |
| 9. |  |  |  |  |  |  | \|__\|__\| | OD / BD / TDS | for \|__\|__\| days |
| 10. |  |  |  |  |  |  | \|__\|__\| | OD / BD / TDS | for \|__\|__\| days |
