# Supplementary material for: Adherence to Dihydroartemisinin + Piperaquine Treatment Regimen in Low and High Endemic Areas in Indonesia
Source: J Trop Med. 2022 Mar 11;2022:4317522. doi: 10.1155/2022/4317522 (PMC8933069; doi:10.1155/2022/4317522)
Supplement: Supplementary Materials — The center questionnaire can be downloaded from Supplementary Material 1. The home questionnaire can be downloaded from Supplementary Material 2. [file 4317522.f1.zip › 4317522.f1/Supplementary 2-Home questionnaire.docx]

**DHP STUDY, INDONESIA**

**Home-Questionnaire**

| **Date: \|__\|__\| (dd) / \|__\|__\| (mm) / 2013** | | | **UNIQUE INCLUSION NUMBER C-\|__\|__\|__\|** | | | | |
| --- | --- | --- | --- | --- | --- | --- | --- |
| INTERVIEWER NAME: __________/ ________/ ________ | | | |  | | | |
| *Interviews need to be conducted ONLY with the patient or caretakers/parents who gave the treatment to the patient (child).*  *If patient or caretakers/parents are not present at your home visit and cannot be traced in a reasonable amount of time consider them as “lost to follow-up”. Select the next home to perform an interview.*  **LOST TO FOLLOW-UP *(circle)*: Yes / No** | | | | | | | |
| *Please use the introduction sheet to explain the purpose of your visit and ask for written consent (separate sheet).*  *If the patient or caretakers/parents refuse to participate, never insist on their participation. Leave the home and select the next home to perform an interview.*  **WRITTEN CONSENT OBTAINED *(circle)*: Yes / No** | | | | | | | |
| I. DEMOGRAPHIC AND SOCIOECONOMIC INFORMATION OF HOUSEHOLD | | | | | | | |
| Q1 | Respondent relationship to patient | Respondent is the patient  Parent (Mother/Father)  Grandparent (Grandfather/Grandmother)  Sibling (Brother/Sister)  Aunt/Uncle  Other (*specify below*)  _______________________________________________ | | | | | 1  2  3  4  5  6 |
| Q2 | Age of patient | \|__\|__\| *years* | | | | | |
| Q3 | Weight of patient | 4.5-8kg (infant)  9-17kg (young children)  18-35kg (adolescent)  >=36kg (adult)  <4.5kg (infant) | | | | 1  2  3  4  5 | |
| Q4 | Sex of patient | Male  Female | | | | | 1  2 |
| Q5 | Respondent him/herself present at the clinic to receive treatment  *If YES, go to Q6* *If NO, go to Q5* | Yes  No | | | | | 1  0 |
| Q6 | *If respondent was not present at clinic:* Who went to the clinic? | Parent (Mother/Father)  Grandparent (Grandfather/Grandmother)  Sibling (Brother/Sister)  Aunt/Uncle  Other (*specify below*)  *____________________________________________________* | | | | | 1  2  3  4  5 |
| Q7 | Patient: highest level of education | Unable to read and write  Primary but incomplete  Primary completed  Secondary but incomplete  Secondary completed  Higher level but incomplete  Higher level completed | | | | | 1  2  3  4  5  6  7 |
| Q8 | How many people live in your household currently?  *Including respondent* | Household members | | | \|__\|__\| | | |
| Q9 | How many children under 5 years of age live in your household? | Children under 5 years | | | \|__\|__\| | | |
| Q10 | What is the profession of the head of household?  *Person who has* ***responsibility*** *for whole* ***household*** | Subsistence farmer  Farmer for trading  Trader  Workman (e.g. joiner, mechanic etc)  Daily worker  No work  Other *(specify below)*  ____________________________________________________ | | | | | 1  2  3  4  5  6  7 |

| II. TREATMENT | | | |
| --- | --- | --- | --- |
| Q11 | How is the patient feeling at the time? *If cured, go to Q12* *If not cured, go to Q11* | Cured / OK  Better but not cured  Still unwell | 1  2  3 |
| Q12 | If patient not cured: Has patient been back to the clinic in the last 2 days? | Yes  No | 1  0 |
| Q13 | Is respondent happy with the treatment received at the clinic? | Yes  No | 1  0 |

| **II.a. TREATMENT INTAKE DAY 0** | | | | | | | | | | | | | |  |  |
| --- | --- | --- | --- | --- | --- | --- | --- | --- | --- | --- | --- | --- | --- | --- | --- |
| ***Show tablet samples to respondent*** | | ASAQ tablets | | | | | | | | | | | |  |  |
| Q14 | While still **at the clinic**, did the patient take any of these tablets? | | | | | | | Yes  No | | | 1  0 | | | |  |
| Q15 | When the patient came **home** from the clinic, did they take any of these tablets before going to sleep? | | | | | | | Yes  No | | | 1  0 | | | |  |
|  | If NO, go to Q19 *If YES, go to Q16* | | | | | | |  | | |  | | |  |  |
| Q16 | How many times total? | \|__\|__\| *times* | | | | | | | | | | | | |  |
| Q17 | How many tablets each time?  *Make an additional note at the back of the paper if more than 3 times* | 1^st^ time | | | | 2^nd^ time | | | 3^rd^ time | | | | | | |
|  |  | \|__\|__\| | | | | \|__\|__\| | | | \|__\|__\| | | | | | | |
| Q18 | When? | Morn  Aft  Eve | 1  2  3 | | | Morn  Aft  Eve | | Morn  Aft  Eve | 1  2  3 | | | Morn  Aft  Eve | | | |
| Q19 | Spat out or vomited tablets? | Yes  No | 1  0 | | | Yes  No | | Yes  No | 1  0 | | | Yes  No | | | |
|  | | | | | | | | | | | | | |  |  |
| **II.b. TREATMENT INTAKE DAY 1** | | | | | | | | | | | | | |  |  |
|  |  |  | | | | | ASAQ tablets | | | | | | |  |  |
| Q20 | The day after the patient went to the clinic did the patient take any tablets? | | | | | | Yes  No | | | | 1  0 | | |  |  |
|  | If NO, go to Q24 *If YES, go to Q21* | | | | | |  | | | |  | | |  |  |
| Q21 | How many times total? | \|__\|__\| *times* | | | | | | | | | | | |  |  |
| Q22 | How many tablets each time?  *Make an additional note at the back of the paper if more than 3 times* | 1^st^ time | | | 2^nd^ time | | | | | 3^rd^ time | | | |  |  |
|  |  | \|_\|_\| | | | \|_\|_\| | | | | | \|_\|_\| | | |  |  |  |
| Q23 | When? | Morn  Aft  Eve | | 1  2  3 | Morn  Aft  Eve | | 1  2  3 | | | Morn  Aft  Eve | | | 1  2  3 |  |  |
| Q24 | Spat out or vomited tablets? | Yes  No | | 1  0 | Yes  No | | 1  0 | | | Yes  No | | | 1  0 |  |  |
|  | | | | | | | | | | | | | |  |  |
| II.c. TREATMENT INTAKE DAY 2 | | | | | | | | | | | | | |  |  |
|  |  |  | | | | | ASAQ tablets | | | | | | |  |  |
| Q25 | What about the following day, did the patient take any tablets? | | | | | | Yes  No | | | | 1  0 | | |  |  |
|  | If NO, go to Q29 *If YES, go to Q26* | | | | | |  | | | |  | | |  |  |
| Q26 | How many times total? | \|__\|__\| *times* | | | | | | | | | | | |  |  |
| Q27 | How many tablets each time?  *Make an additional note at the back of the paper if more than 3 times* | 1st time | | | | 2nd time | | | 3rd time | | | | | | |
|  |  | \|__\|__\| | | | | \|__\|__\| | | | \|__\|__\| | | | | | | |
| Q28 | When? | Morn  Aft  Eve | 1  2  3 | | | Morn  Aft  Eve | | 1  2  3 | Morn  Aft  Eve | | | 1  2  3 | | | |
| Q29 | Spat out or vomited tablets? | Yes  No | 1  0 | | | Yes  No | | 1  0 | Yes  No | | | 1  0 | | | |

| III. PRESENCE OF MEDICINE | | | | | |
| --- | --- | --- | --- | --- | --- |
| Q30 | Ask to see the medicine/ medicine packaging given to them at the clinic | Empty ACT or primaquine blister / labelled drug bag seen  Blister / drug bag with tablets seen  No blister / bag seen | | 1  2  0 | |
|  | | | | | |
| *If tablets still present*  *If all tablets taken but incorrectly*  *If all tablets taken correctly*  *If blister / bag was NOT seen:*  *Check if tablets were taken incorrectly*  *Check if tablets are taken correctly* | | | *Go to Q31*  *Go to Q33*  *Go to Q34*  *Go to Q33*  *Go to Q34* | | |
| Q31 | How many tablets are remaining?  *Must be* ***seen*** *by interviewer* | Number of tablets seen: | \|__\|__\| | | |
| Q32 | Can you tell me why there are tablets remaining?  *Multiple answers possible, please circle* ***all*** *responses given*  *Now go to Q34* | Patient got cured, so no need to continue the treatment  Patient got cured, so I kept the remaining tablets for next time  Household poor, so kept some tablets for next time  Patient forgot to take some tablets / Caretaker forgot to give some tablets  Patient did not feel better, the treatment was not working  Patient / Caretaker claims that wrong instructions were given in the clinic  Other reasons/s (*specify below)*  *______________________________________________________________*  *______________________________________________________________* | | | 1  2  3  4  5  6  7 |
| Q33 | The patient has taken the tablets incorrectly, why?  *Multiple answers possible, please circle* ***all*** *responses given*  *Now go to Q34* | Patient / Caretaker thought patient would cure faster  Patient / Caretaker claims that wrong instructions were given in the clinic  Tablets made patient feel sick  Patient could not swallow tablets  Patient was vomiting  Other reason/s (*specify below)*  *______________________________________________________________*  *______________________________________________________________* | | | 1  2  3  4  5  6 |
| Q34 | The tablets have been taken correctly, well done! Why do you think it was done correctly?  *Multiple answers possible, please circle* ***all*** *responses given* | Patient / Caretaker / household member previsouly taken / given the same medicine before and knows how to take it  Right instructions were given at the clinic  Was helped by the community health volunteer (CHV)  Other reason/s (*specify below)*  *______________________________________________________________*  *______________________________________________________________* | | | 1  2  3  4 |
| Q35 | In the past 3 days, did the patient take any other medication to treat malaria?  *If YES go to Q35*  *If NO go to Q37* | Yes  No | | | 1  0 |
| Q36 | If yes, what was taken?  *Show respondent tablet samples*  *Multiple answers possible, please circle* ***all*** *responses given* | Paracetamol (PCM)  Folic Acid (FA)  Herbal *(specify below)*  *______________________________________________________________*  Other/s *(specify below)*  *______________________________________________________________* | | | 1  2  3  4 |
| Q37 | If yes, where did this medicine come from?  *Multiple answers possible, please circle* ***all*** *responses given* | Clinic during same consultation  Other public health structure *(specify i.e. Gov. hospital, PHU, CHV, GRC)*  *______________________________________________________________*  Home / Relative / Friend  Bought on market  Traditional healer  Other place/s *(specify below)*  *______________________________________________________________* | | | 1  2  3  4  5  6 |

| **IV. PERCEPTION OF MALARIA** | | | |
| --- | --- | --- | --- |
| Q38 | The patient had malaria. Do you know how you get sick with malaria?  *Multiple answers possible, please circle* ***all*** *responses given* | Don’t know  Standing/ working/ playing in hot sun  Bad spirit  Contaminated water  Problem with food  Mosquito bite  Other reason/s *(specify below)*  *______________________________________________________________*  *______________________________________________________________*  *______________________________________________________________* | 1  2  3  4  5  6  7 |
| Q39 | How do you think you can protect yourself from getting malaria?  *Multiple answers possible, please circle* ***all*** *responses given* | Don’t know  Sleep under mosquito net  Amulettes or magic  Taking medication  Cleaning around the house  Good cooking habits  Spraying inside the house  Digging a latrine  Other protection/s (*specify below)*  *______________________________________________________________*  *______________________________________________________________*  *______________________________________________________________* | 1  2  3  4  5  6  7  8  9 |
| Q40 | How many bednets are present in your house?  *If none, please enter ‘0’* | \|__\|__\| bednets | |
| Q41 | Do you know what ACT use for?  *Multiple answers possible, please circle* ***all*** *responses given* | Don’t know  To treat malaria  To cure fever and headache  To make the body feel better  Other protection/s (*specify below)*  *______________________________________________________________*  *______________________________________________________________*  *______________________________________________________________* | |
| Q42 | Do you know what primaquine use for?  *Multiple answers possible, please circle* ***all*** *responses given* | Don’t know  To treat malaria  To cure fever and headache  To make the body feel better  To prevent malaria transmission  Other protection/s (*specify below)*  *______________________________________________________________*  *______________________________________________________________*  *______________________________________________________________* | |

**Please thank the respondent very much for his/her participation and help.**
